# The ability of the geriatric nutritional risk index to predict the risk of heart diseases in Korean adults: a Korean Genome and Epidemiology Study cohort

**DOI:** 10.3389/fnut.2023.1276073

**Published:** 2023-10-26

**Authors:** Ju Young Park, So Young Bu

**Affiliations:** Department of Food and Nutrition, Daegu University, Gyeongsan, Republic of Korea

**Keywords:** malnutrition, geriatric, KoGES, heart, aging

## Abstract

**Introduction:**

The predictive ability of nutritional risk index on cardiovascular outcomes in middle-aged and non-hospitalized adults has not yet been reported. This study investigated whether the Geriatric Nutritional Risk Index (GNRI), an index for assessing the risk of developing malnutrition, could predict heart disease in middle-aged Korean adults.

**Methods:**

The cohort used in this study consisted of 3,783 participants selected from 10,030 Korean adults who participated in the Ansan-Ansung cohort study as part of the Korean Genome and Epidemiology Study. The GNRI was determined based on serum albumin level, proportion of current weight, and ideal body weight. Participants were then divided into two groups: GNRI ≤98 and > 98, which corresponded to the risk of malnutrition and normal, respectively. The major outcome of this study was coronary artery disease (CAD) or congestive heart failure (CHF) during a 15-year-follow period.

**Results:**

During the follow-up period spanning 2004–2018, 136 events of heart disease occurred. Using a Kaplan–Meier analysis, event-free rates were found to be associated with 90.5% on a GNRI ≤98 and 96.6% on a GNRI >98 (*p* < 0.0009). GNRI ≤98 showed a 3.2-fold (hazard ratio, 3.22; 95% credit interval, 1.49–6.96; *p* = 0.0029) increase in the incidence of heart disease, including CAD or CHF, compared with GNRI >98, after controlling for potential confounders.

**Conclusion:**

Malnutrition risk confers a significantly increased risk for heart disease in middle-aged Koreans. Further studies with larger cohorts are needed to verify the efficacy of the GNRI in predicting disease risk in adults with pre-disease.

## 1. Introduction

As the older population increases, health problems specific to the aging process and nutritional issues are receiving increasing attention. Nutritional disorders, due to either excess nutrient intake or deficiency, affect the development of diseases in older adults and patients with several diseases (1, 2). In addition, diseases induced by malnutrition tend to have different aspects from those induced by overnutrition, which are usually obesity-related complications such as hypertension, diabetes, and dyslipidemia (2). Malnutrition or the risk factors for malnutrition can lead to the deterioration of body composition, loss of skeletal and cardiac muscles (3, 4), and compromised immune function (5, 6). To date, the health consequences of malnutrition have primarily been investigated in hospitalized patients with several diseases, because disease status leads to nutritional deficiency. Indeed, a large proportion of patients with end-stage diseases (e.g., cancer or renal disease) are malnourished (3) and exhibit impaired immune function, attenuated wound healing, and disease aggravation (5, 7–9). In particular, malnutrition has been associated with an increased mortality rate in patients with coronary heart disease, heart failure, and older adults (10–12). It has also been associated with the incidence of coronary heart disease in several cross-sectional studies (13, 14). Malnutrition has also been reported at a substantial rate in young and externally healthy individuals with potential health risk factors (15–17). Although obesity has been investigated in various populations, including younger adults, its consequences in several contexts remain to be investigated.

The Geriatric Nutritional Risk Index (GNRI) is an screening tool of nutrition-related risk for estimating the likelihood of morbidity and mortality in older populations and chronically ill patients (14, 18–21). The GNRI comprises two parameters, body weight and serum albumin, which are simple to measure and obtain from routine check-ups in hospitals and community-based health centers (22). Community-based studies have found that a low GNRI indicating the risk of malnutrition is associated with a higher mortality risk due to heart failure (14, 18, 19). This index is usually applied to groups of patients or older adults, mostly those aged 65 years and older. Owing to the aging population in Korea, the number of patients with cardiovascular diseases (CVDs) and heart failure is increasing in Korea. The estimated heart failure rates were 0.77% in 2002 and 2.24% in 2018 (23). The prevalence of heart failure is 0.1–0.7% in young and middle-aged adults aged 50 years or younger, but this number increases to 16.9% in later life (23). In most previous studies, the risk of malnutrition and its outcomes were primarily investigated in older populations, usually those aged 60 years and above (12). These reports, in turn, indicate that malnutrition, which has already been initiated in midlife, affects health outcomes later in life. However, little is known regarding the association between GNRI-assessed the risk of malnutrition and harmful cardiac events in middle-aged adults. Although the main population for assessing the prognostic efficacy of the GNRI is the older population (24), the GNRI has been validated in young adults (25, 26).

Hence, the prognostic efficacy of the GNRI in predicting the occurrence of cardiovascular and heart diseases in conjunction with the aging process should be investigated earlier in an individual’s life than previously reported. In addition, few studies have evaluated the link between malnutrition and various cardiovascular complications in a cohort of hospitalized patients (27); however, its consequences in the general population need to be assessed. Thus, the present study aimed to examine whether the GNRI is a valid predictor of heart disease in middle-aged Korean adults.

## 2. Study population and methods

### 2.1. Study participants

**Figure 1:**
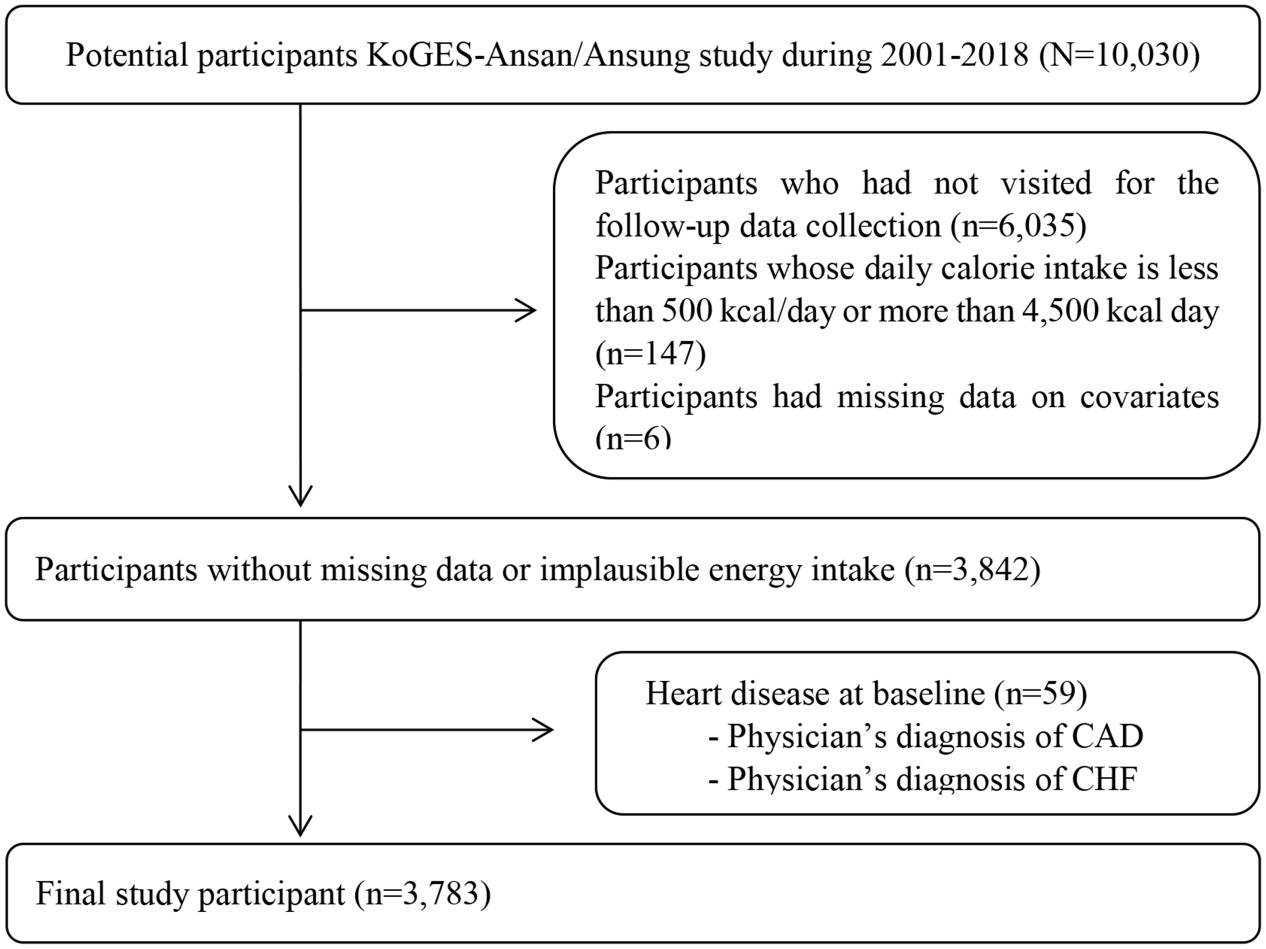
Flow diagram for selecting study participants. The requirement of obtaining consents was waived by the Institutional Review Board. KoGES, The Korean Genome and Epidemiology Study; CAD, coronary artery disease; CHF, congestive heart failure.

Participants were enrolled from two community-based cohorts, the Ansung and Ansan cohorts, from the Korean Genome and Epidemiology Study (KoGES) (28). The eligibility criteria for participating in the KoGES were 40–69 years of age and dwelling within the community for 6 months or longer by the time of enrolment. The participants voluntarily enrolled in the study and provided written informed consent. Detailed information on the KoGES design, processes, and participant retention rates has been published previously (28). At the beginning of the study, 10,030 participants were included in KoGES. Baseline measurements were conducted in 2001 and 2002, and biannual follow-up examinations were conducted until 2018. We excluded participants who had not visited for follow-up data collection (*n* = 6,035), had a total energy intake value <500 kcal/day, or ≥ 4,500 kcal/day (*n* = 147), had missing data (*n* = 6), or already had coronary artery disease (CAD) and congestive heart failure (CHF) at baseline data collection (*n* = 635). Finally, 3,783 participants were included in the analysis. The procedure for selecting the participants is shown in Figure 1. This study was approved by the institutional review board (IRB) of Daegu University. In addition, the personal identifying information of the study participants was deleted from the dataset prior to acquisition and analysis. The requirement for written informed consent from KoGES study respondents was waived by the IRB of Daegu University.

### 2.2. Data collection

Demographic information was collected using a standard questionnaire administered during in-person interviews at the KoGES (28). Demographic data included age, sex, and educational level. Body weight, height, BMI (kg/m^2^), muscle mass (kg), systolic blood pressure (SBP), diastolic blood pressure (DBP), waist circumference, alcohol intake (currently drinking alcohol), and smoking status (currently smoking cigarettes) were recorded. Body fat and muscle mass were measured using bioelectrical impedance analysis (Biospace, Seoul, Korea) (29). For analysis of GNRI distribution according to BMI criteria, BMI was categorized as either “underweight (BMI < 18.5),” “normal (18.5 ≤ BMI <23),” “overweight (23 ≤ BMI <25),” or “obese (25 ≤ BMI)” according to the BMI classification for Asian and South Asian population suggested by the National Institute of Health (30). Peripheral blood samples were drawn from the study participants (28). Albumin, high-sensitivity C-reactive protein (hs-CRP), triglyceride, total cholesterol, high-density lipoprotein cholesterol (HDL), fasting glucose, hemoglobin A1c (HbA1c), blood urea nitrogen (BUN), and creatinine were used as covariates in this study. Hypertension was defined as SBP ≥ 140 mmHg or DBP ≥ 90 mmHg, antihypertensive medication use, or a diagnosis made by a medical doctor. Diabetes mellitus (DM) was determined as fasting blood glucose ≥126 mg/dL, HbA1c ≥ 6.5%, antidiabetic treatment use, or a physician’s diagnosis. The diagnosis of CAD or CHF, the main outcome of this study, was reported using a questionnaire administered during the KoGES study period. To estimate nutritional intake status, dietary data were collected using a Korea-specific food frequency questionnaire that asked about the frequency and portion size of each food item consumed by each participant during the last year. Daily total nutrient intake was assessed by applying the amount and type of each reported food item to the CAN-Pro 2.0 program developed by the Korean Nutrition Society (31).

### 2.3. Geriatric nutritional risk index

The GNRI was calculated using the following formula: GNRI = (1.489 × serum albumin [g/L]) + (41.7 × weight [kg]/ideal body weight [kg]). Ideal body weight was determined as 22 × the square of height, as previously described (20, 24, 26). The variables used in this formula were assessed during the baseline visit to KoGES. Previously, GNRI values were divided into three categories: < 82, < 92, 92–98, and > 98 for major, moderate, low and no “nutrition-related risk,” respectively (21), and into two categories: GNRI <98 and GNRI >98, indicating malnutrition and adequate nutrition, respectively (20). Because the number of participants categorized as 92 < GNRI ≤98, and GNRI <92 was small (less than 5% of total participants) in this study, the presence of malnutrition risk based on GNRI was categorized into two levels: GNRI ≤98; and GNRI >98. Recent studies have shown that the GNRI is applicable to participants regardless of their body fluid status or disease type. Hence, this study did not limit the study participants owing to the presence of disease status.

### 2.4. Statistical analysis

All data analyses were performed using the SAS software (SAS 9.4; SAS Institute Inc., Cary, NC, United States). Continuous variables are expressed as mean ± standard deviation, and categorical variables are expressed as numbers and percentages in parentheses. Differences in variables between the groups were evaluated using analysis of variance for continuous variables and the chi-squared test for categorical variables. Fisher’s exact test was used when the cell count per event was <5. In this study, multivariate regression analysis was performed to assess the contribution of the measured variables to GNRI values. Kaplan–Meier curves were plotted from the reported date of the diagnosis of heart disease (CAD or CHF) and compared between groups with GNRI ≤98 and GNRI >98. A Cox proportional hazards regression model was used to test the association between the risk of malnutrition based on the GNRI score and the incidence of heart disease. Model 1 was a crude model that only assessed the association between the incidence of heart disease and GNRI, and Model 2 was further adjusted for age, sex, and BMI. Model 3 included the same variables as Model 2 and cigarette smoking status, current alcohol consumption, and educational level. Model 4 added the following variables to Model 3: the presence of hypertension, DM, hyperlipidemia, and any use of medication for these diseases. Statistical significance was confirmed at *p*-values <0.05.

## 3. Results

### 3.1. Baseline characteristics of the study participants between GNRI categories

The baseline characteristics of the study participants are presented in Table 1. The number of participants in GNRI ≤98 and > 98 was 105 and 3,678 patients, respectively. At the initial examination, the ages of the two groups were 55.1 and 50.8 years. The proportion of adults aged 40–50 years among normal participants was the highest among all age groups, while the proportion of adults aged 60–69 years was the highest among participants with GNRI ≤98. Except for the height variable, the values of all anthropometric parameters, body weight, BMI, muscle mass, and waist circumference were lower in participants with GNRI ≤98 than in those with GNRI >98 (*p* < 0.0001 for all). The SBP was not significantly different between the two groups, and the DBP was lower in the malnourished group than in the normal group (*p* = 0.0033). HDL-cholesterol was the only biochemical parameter higher in participants with GNRI ≤98 than in those with GNRI >98. The levels of serum albumin, triglycerides, total cholesterol, fasting glucose, BUN, and serum creatinine were significantly lower in participants with GNRI ≤98 than in those with GNRI >98. No significant differences were found in the HbA1c or hs-CRP levels. Participants with GNRI >98 tended to have higher proportions of hypertension and hyperlipidemia and medication use for treating these diseases. In contrast, participants with GNRI ≤98 tended to have a lower education level and a higher proportion of individuals who currently smoke cigarettes than those with GNRI >98. Table 2 presents the participants’ intake statuses. The intake levels of total energy, protein, fat, carbohydrates, calcium, and iron were not significantly different between the two GNRI groups. Participants with GNRI ≤98 ate less sodium but took more energy from alcohol than those with GNRI >98. There were no significant differences in the distribution of participants who consumed total daily energy <75%, > 125%, or 75–125% range of the dietary reference for Koreans.

**Table 1:** Baseline characteristics of all the subjects between GNRI categories.

	GNRI ≤98 (*n* = 105)	GNRI >98 (*n* = 3,678)	*p* value
Age	55.1 ± 9.1^*^	50.8 ± 7.9	< 0.0001
40 ≤ year<50	34 (32.4)^†^	1,931 (52.5)	< 0.0001
50 ≤ year<60	32 (30.5)	1,068 (29.0)	
60 ≤ year<70	39 (37.1)	679 (18.5)	
Sex			
Men	62 (59.1)	1,687 (40.9)	0.0076
Women	43 (45.9)	1,991 (54.1)	
GNRI	95.3 ± 2.4	111.8 ± 7.1	< 0.0001
Body weight (kg)	49.8 ± 5.6	63.9 ± 9.7	< 0.0001
Height (cm)	160.6 ± 8.1	160.2 ± 8.5	0.6172
BMI (kg/m^2^)	19.3 ± 1.3	24.8 ± 3.0	< 0.0001
Muscle mass (kg)	38.2 ± 5.6	43.9 ± 8.0	< 0.0001
Waist circumference (cm)	72.2 ± 5.8	82.9 ± 8.5	< 0.0001
SBP (mmHg)	117.4 ± 17.5	120.1 ± 17.4	0.1222
DBP (mmHg)	76.7 ± 10.8	79.9 ± 11.1	0.0033
Albumin (g/L)	3.9 ± 0.2	4.3 ± 0.3	< 0.0001
Triglycerides (mg/dL)	120.6 ± 47.7	161.2 ± 102.0	< 0.0001
Total Cholesterol (mg/dL)	157.7 ± 28.9	191.7 ± 34.0	< 0.0001
HDL-Cholesterol (mg/dL)	47.6 ± 9.9	44.4 ± 9.8	0.0012
Fasting glucose (mg/dL)	80.9 ± 22.3	86.5 ± 19.2	0.0037
HbA1c (%)	5.6 ± 0.9	5.7 ± 0.8	0.2775
hs-CRP (mg/L)	0.27 ± 0.56	0.21 ± 0.57	0.3750
BUN (mg/dL)	13.5 ± 3.4	14.3 ± 3.6	0.0284
Creatinine (mg/dL)	0.8 ± 0.1	0.8 ± 0.2	0.0005
Hypertension (%)	2 (1.9)	521 (14.2)	< 0.0001
Diabetes mellitus (%)	3 (2.9)	189 (5.1)	0.4918
Hyperlipidemia (%)	9 (8.6)	805 (21.9)	0.0011
Medication (%)	1 (1.0)	405 (11.0)	0.0002
Education			
Above high school	73 (69.5)	1,886 (51.3)	0.0002
Below high school	32 (30.5)	1,792 (48.7)	
Current alcohol drinker	55 (52.4)	1,770 (48.1)	0.3894
Current smoker	43 (41.0)	775 (20.5)	< 0.0001

^*^Mean ± S.E., ^†^number of frequency (%), the *p* values are from ANOVA for continuous variables and Chi square test for categorical variables for assessing the trend of difference between GNRI categories.BMI, body mass index; SBP, systolic blood pressure; DBP, diastolic blood pressure; hs-CRP, high sensitive C-reactive protein; BUN, blood urea nitrogen. Medication: any medication against for diabetes, hypertension and hyperlipidemia.

**Table 2:** Nutrient intake status of study participants.

	GNRI ≤98 (*n* = 105)	GNRI >98 (*n* = 3,678)	*p*
Energy (kcal)	1,961.4 ± 661.3^*^	1,936.3 ± 588.0	0.6677
Protein (g)	65.2 ± 29.3	65.3 ± 24.3	0.9578
Fat (g)	31.1 ± 20.0	31.9 ± 17.4	0.6600
Carbohydrate (g)	349.9 ± 113.5	342.0 ± 102.8	0.4400
Calcium (mg)	451.8 ± 241.9	472.2 ± 250.4	0.4105
Iron (mg)	10.8 ± 5.1	10.8 ± 4.6	0.9153
Sodium (mg)	3,457.1 ± 1,982.5	3,132.1 ± 1,540.9	0.0348
Energy intake from alcohol	90.6 ± 186.6^‡^	61.4 ± 140.5	0.0413
Alcohol/energy ratio	4.7 ± 9.6	3.2 ± 7.4	0.0465
Carbohydrate/energy ratio	72.2 ± 7.7	71.1 ± 6.6	0.0970
Protein/energy ratio	13.1 ± 2.8	13.4 ± 2.2	0.1203
Fat/energy ratio	13.6 ± 5.8	14.4 ± 5.2	0.1292
Energy intake status			
<75% KDRI	28 (26.7)^†^	934 (25.4)	0.5962
75% ≤ KDRI ≤125%	59 (56.2)	2,194 (59.7)	
> 125% KDRI	18 (17.1)	550 (15.0)	

^*^Mean ± S.E., ^†^Percent intake from total energy intake, ^‡^Caloric intake from alcohol was calculated by g of alcohol intake/day × 7.0 kcal/g of alcohol.The *p* values are from ANOVA or Chi square test for assessing the trend of difference between GNRI categories. KDRI: 2015 Dietary reference intake for Koreans.

### 3.2. Ratio of nutritional risk in participants stratified by BMI

As BMI has been previously used to assess malnutrition status, the study participants were categorized into four weight statuses according to BMI, and the distribution of BMI was compared between the GNRI groups (Table 3). Participants with GNRI ≤98 comprised only underweight and normal individuals without overweight or obese participants. Participants with GNRI >98 had the highest proportion of individuals who were “obese” (45%), and < 30% of participants were “underweight” or “normal” based on BMI criteria.

**Table 3:** Prevalence of GNRI-assessed malnutrition risk according to BMI of participants (*n* = 3,783).

	Underweight	Normal	Overweight	Obese	*p*-value^*^
Malnourished (GNRI ≤98)	27 (25.7)	78 (74.3)	0 (0.00)	0 (0.00)	< 0.0001
Normal (GNRI >98)	18 (0.5)	990 (26.9)	1,016 (27.6)	1,654 (45.0)	
Total	45	1,068	1,016	1,654	

Data are presented as *n* (%).^*^Fisher’s exact test was performed to compare the distribution of BMI between malnourished and normal.GNRI: geriatric nutritional risk index; Underweight: BMI < 18.5, Normal: 18.5 ≤ BMI <23, Overweight: 23 ≤ BMI <25, Obese: 25 ≤ BMI.

### 3.3. Exploring the variables that contribute to the GNRI

The linear association between the GNRI values and several baseline variables was explored to investigate how the GNRI values were affected (Table 4). The GNRI value was significantly associated with age (β = −0.08, *p* < 0.0001), BMI (β = 1.97, *p* < 0.0001), sex (β = −1.05, *p* < 0.0001), hs-CRP (β = −0.33, *p* = 0.0079), current smoking status (β = −1.12, *p* < 0.0001), total energy intake (β = −0.06, *p* < 0.0001), and the level of education (β = 0.83, *p* < 0.0001). No significant association with the GNRI was found for disease status, including hypertension, diabetes, and hyperlipidemia, or the use of medication against these diseases.

**Table 4:** Relationship between GNRI and variables with multivariate regression analyses.

**Model fit**		
R2	0.6622	< 0.0001^*^
Intercept	68.48	< 0.0001
**Parameter estimates**		
	*β*	
Age	−0.08	< 0.0001
BMI	1.97	< 0.0001
Sex	−1.05	< 0.0001
hs-CRP	−0.33	0.0079
Smoke	−1.12	< 0.0001
Drink	0.17	0.3005
Energy intake	−0.06	< 0.0001
Education	0.83	< 0.0001
Hypertension	0.60	0.0719
Diabetes mellitus	−0.49	0.1383
Hyperlipidemia	0.17	0.6934
Medication	−0.12	0.7408

*The *p*-value was determined with multivariate regression analysis.BMI, body mass index; hs-CRP, C-reactive protein; GNRI, geriatric nutritional risk index.

### 3.4. Accumulated heart disease incidence according to GNRI values during the follow-up period

**Figure 2:**
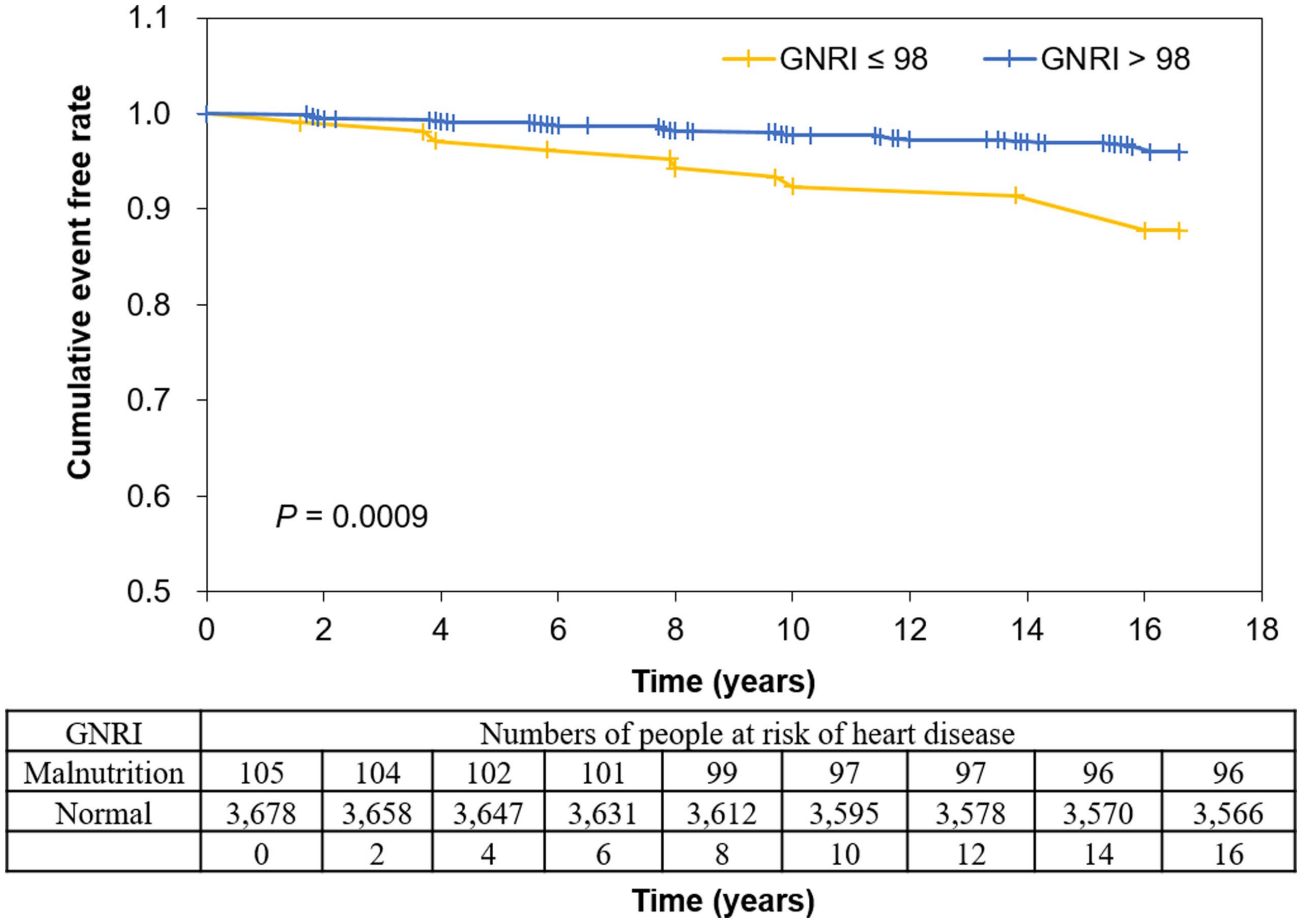
Kaplan–Meier curves for events of heart disease according to GNRI. Disease occurrence was negatively associated with GNRI, with a lower diagnostic-free curve for GNRI ≤98. Log-rank test indicated *p* = 0.0009. GNRI: geriatric nutritional risk index.

The event rate of heart disease during the 15-year follow-up period was 3.60% (*n* = 136 events) in all participants (Table 5). The ratios of diagnosed disease in GNRI ≤98 and GNRI >98 were 9.5% and 3.4% for total heart disease (*p* = 0.0009), 8.6% and 3.3% for CAD (*p* = 0.0037), and 1.0% and 0.2% for CHF (*p* = 0.1790), respectively. In Kaplan–Meier curves, participants in GNRI ≤98 showed a significantly higher incident probability of heart diseases compared with those in GNRI >98 (log-rank test *p* value = 0.0009) (Figure 2).

**Table 5:** The prevalence of heart disease during 15 year-follow-up period.

Events	*N* (%)		
	GNRI ≤ 98 (*n* = 105)	98 < GNRI (*n* = 3,678)	*p*-value
Total CC events, *n* (%)	10 (9.5)	126 (3.4)	0.0009
Coronary artery disease, *n* (%)	9 (8.6)	122 (3.3)	0.0037
Congestive heart disease, *n* (%)	1 (1.0)	6 (0.2)	0.1790^*^

The prevalence of cardiac complications was examined between participants with low GNRI and high GNRI, and the difference of prevalence was analyzed by Chi-square analysis.^*^Fisher’s exact test was performed when the cell counts for each event was less than 5.GNRI, geriatric nutritional risk index.

### 3.5. Association between GNRI and the incidence of heart diseases

Table 6 shows the results of the Cox hazard regression analysis to determine the risk of heart disease based on GNRI score. The participants with GNRI ≤98 showed an increased heart disease risk compared to those with GNRI >98. The crude hazard ratios (95% credit interval, CI) for diagnosis of heart disease without adjustment (Model I) in GNRI ≤98 relative to GNRI >98 was 2.83 (1.49–5.40) (*p* for trend = 0.0015), respectively. The degree of risk of heart disease in the participants with GNRI ≤98 to the participants with GNRI >98 remained similar after gradual adjustment of age and height (Model II, *p* for trend = 0.0014), the level of education, smoking and alcohol consumption, total energy intake, sodium intake (Model III, *p* for trend = 0.0018), and history of diabetes or hyperlipidemia and medication for diabetes and hyperlipidemia (Model IV, *p* for trend = 0.0029) in various regression models.

**Table 6:** Cox regression analyses for heart disease.

	Hazard ratio (95% CI)		
	GNRI ≤ 98 (*n* = 105)	98 < GNRI (*n* = 3,678)	*p*-value
Model I	2.83 (1.49–5.40)	1.00 (reference)	0.0015
Model II	3.30 (1.58–6.85)	1.00 (reference)	0.0014
Model III	3.23 (1.54–6.76)	1.00 (reference)	0.0018
Model IV	3.22 (1.49–6.96)	1.00 (reference)	0.0029

Hazard ratio with 95% confidence interval were shown.Model I: crude model, Model II: adjusted for age, BMI, and sex, Model III: Model II + the degree of education + household income + smoke + drink. Model IV: Model III + diabetes mellitus + hypertension + dyslipidemia + any medication for diabetes, hypertension and hyperlipidemia.

## 4. Discussion

The present study reported an association between the GNRI and the risk of heart disease in middle-aged Korean adults. The results showed that adults with malnutrition risk, as judged by the GNRI, had an increased risk of developing heart diseases, including CVD and CHF, compared to adults with no risk. In this study, various covariates were related to the GNRI, but the significance of the association remained even after adjusting for several covariates. To the best of our knowledge, this is the first study to use the GNRI to predict heart disease in middle-aged Korean adults.

The GNRI has long been used to assess the nutrition-related risk of hospitalized patients and outpatients with various diseases (19, 32). The GNRI mainly comprises serum albumin and body weight, which are regularly assessed in hospitalized patients or the diagnostic procedure of outpatients (22). Serum albumin and BMI are typical indicators of nutritional status and are used to predict mortality and disease-related complications in patients with CAD (33, 34). Heart disease is closely related to malnutrition (4, 10, 12). The relationship between malnutrition and heart disease has been reported in several cross-sectional cohort studies. For instance, malnourished individuals categorized by serum albumin levels showed increased left ventricular mass index and ventricular wall thickness and decreased diastolic function in the general Taiwanese population (35). Another study reported that low muscle mass and strength are associated with heart failure in hospitalized older patients with physical disabilities (36). Under malnutrition status, including weight loss of >5% within the past 6 months, a BMI < 22 and muscle indices were highly correlated with the relative risk of cardiovascular complications, including arrhythmia, myocardial infarction, and CHF in older adult patients (37). In addition, differences in the nutritional status of patients with CVD vary according to sex. In patients with acute myocardial infarction, the risk of malnutrition screened by the “Nutritional Risk Screening 2002 (NRS-2002)” significantly predicted in-hospital mortality in female patients but not in male patients (38) indicating the possibility of malnutrition as a sex-specific factor in predicting disease risk or mortality. Recent studies have indicated that the risk of malnutrition assessed by the GNRI accurately predicts disease risk and cardiovascular mortality (20, 39–41), and these findings appear consistent with those of a previous study assessing a single individual indicator of malnutrition (22, 33, 34). Furthermore, GNRI was associated with all-cause mortality in the general population, including healthy adults, in a national health and nutrition survey conducted in the USA (42). Although participants with GNRI ≤98 all belonged to underweight or normal based on BMI in this study, BMI alone did not predict the risk of heart disease (data are not shown). In addition, the low GNRI group tended to have low fat and muscle mass along with decreased body weight, indicating an overall decline or deterioration in body composition under the conditions of developing malnutrition. In line with a previous report (39), our results indicate that the GNRI is a more dependable index than serum albumin or BMI alone for predicting heart disease in Korean adults.

Most patients with heart disease have multiple comorbidities, are vulnerable to infections or unexpected communicable diseases, and face challenges in clinical management (43–45). Although heart disease does not cause mortality, it is considered life-threatening. According to the Health Insurance Review and Assessment Service in Korea, the number of malnourished patients aged 20 years and diagnosed with malnutrition was 149 and 791, respectively, in 2017 and 335 and 441, respectively, in 2021, which was a 23.9% increase over the last 5 years. Of these, 29.4% were adults in their 60s, with the highest prevalence; however, the prevalence in adults in their 40s and 50s was 20 and 21.9%, respectively (46), indicating that malnutrition begins at an earlier age than the age at which the consequences of malnutrition occur. The age range of the participants was 40–69 years at baseline. Although the GNRI ≤98 group had a higher proportion of older adults aged ≥60 years than the GNRI >98 group, the proportion of participants aged 40–50 years was more than 30% within the group with GNRI ≤98. Studies have also suggested that young and middle-aged adults have nutritional deficiencies and a potential risk of heart failure and malnutrition (47, 48). Although previous studies have focused excessively on malnutrition in the aged population (20, 24, 39), earlier identification of malnutrition can advance disease treatment and recovery. Hence, our findings indicate the need to identify nutrition-related risk in middle-aged adults to prevent cardiovascular risks later in life.

Regarding the findings of this study, there are possible mechanisms through which risk factors developing malnutrition affects heart disease. Malnutrition hinders the recovery from inflammatory conditions due to disease or infection and continued inflammatory conditions, leading to catabolic reactions that promote protein degradation and muscle wasting (49, 50). In addition, the malnutrition status of the study cohort coincided with high levels of inflammatory mediators such as tumor necrosis factor-alpha (51, 52) and hs-CRP, which are highly correlated with the risk of CVD and heart arrest (18, 50). In line with these findings, hs-CRP was negatively correlated with GNRI score in the regression model adjusted for several covariates in this study. Previously, hs-CRP was associated with atherosclerosis in adults aged 50–64 years (53). Serum albumin, the main component of the GNRI, is closely associated with hs-CRP levels in patients with inflammatory diseases. For instance, patients with higher albumin tend to have higher triceps skinfold measurements reflecting upper arm muscle circumference and lower hs-CRP levels in those with Crohn’s disease (54). Moreover, malnutrition is known to weaken immune function owing to the loss of body proteins and energy restriction (55). In addition, sarcopenia is associated with atherosclerosis and impaired endothelial function in older populations (56). Muscle wasting is associated with increased arterial stiffness and risk of CVDs in middle-aged adults (57). Therefore, a positive feedback loop may have existed between inflammation, malnutrition, cardiac muscle weakening, and adverse cardiac events in the study cohort.

In this study, the total calorie and other nutrient intakes at baseline were not significantly different between the GNRI >98 and GNRI ≤98 groups. However, calorie intake from alcohol consumption was significantly higher in participants with GNRI ≤98 and led to a higher ratio of energy from alcohol to total energy intake than in those with GNRI >98. Alcohol intake has been known to be associated with heart failure, cardiomyopathy, and cardiomyopathy-derived mortality and worsens the status of malnutrition (58, 59). Although alcohol consumption in the study participants was approximately 13.0 g per day, a recent study reported that an increase in alcohol consumption was linearly associated with the risk of CAD (60). One study showed that an increase in alcohol consumption increased the risk of CAD by 1.4-fold compared with non-alcohol consumption (60), indicating that habitual alcohol intake increases the risk of heart disease, even in low amounts. Because this study investigated the nutritional intake data of participants only available at baseline, the change in their diet, including nutrient intake, with regard to the incidence of heart disease, needs to be further investigated in a future study. Nevertheless, our data suggest that identifying nutrition-related risk and early nutritional intervention at the earliest possible time could reduce the risk of disease-related complications. According to the American Heart Association and Heart Failure Society guidelines, evaluation of nutritional status is recommended for patients with heart failure (61). In addition, studies have reported that nutritional intervention may improve clinical outcomes in older adults aged ≥60 years (62, 63). A randomized clinical trial and a cross-sectional study of community-dwelling older adults found that supplementing or enhancing their energy status and protein consumption improved their nutritional status (63, 64) and reduced disease prevalence (65). Moreover, sex differences exist in the association of malnutrition with heart disease (38) and cardiovascular outcomes in response to nutritional supplements or dietary patterns of patients (66). However, discrepant results indicate that being underweight and the risk of malnutrition are directly associated with the odds of in-hospital mortality in men but not in women (67). Based on previous studies and reports and the findings of this study, further long-term research is warranted to identify the main modifiable factors that enhance nutritional status assessed by the GNRI, including sex differences, and to confirm whether nutritional interventions intended to enhance the GNRI of malnourished adults provide health benefits later in life.

According to previous studies, the relationship between the GNRI and the HR of all-cause death or disease incidence was not linear. The curve of the plotted GNRI tended to be flat when the GNRI value exceeded 98, which is the cutoff value used in most previous studies (19, 20, 39). This study also did not observe an association when a GNRI cutoff value >98 was used to categorize the participants (data not shown). This suggests that the incidence of cardiac events does not change when the GNRI exceeds a specific threshold. These results may be explicated by the fact that obesity is a risk factor for CAD in relation to the double burden of malnutrition and obesity (5, 15). Hence, malnutrition, not just an absolute deficit in total energy consumption, but in the context of nutrient balance, and its consequences in heart disease need to be further investigated.

This study had several limitations. First, the sample size of the patient cohort was small after applying the exclusion criteria, and unknown confounding factors may have affected the outcomes. Second, only one nutritional risk index (GNRI) was used to screen the nutritional risk of the participants; hence, the data need to be validated using other nutritional assessment tools. Third, the main outcome–the diagnosis of heart disease–was based only on the participants’ responses to the questionnaire rather than on an objective assessment. However, the percentage of CAD reported within this study cohort was similar to the statistics reported in the fact sheet provided by the Korean Society of Heart Failure (23) and health checkup questionnaire procedures were conducted by well-trained personnel in this nationwide cohort study (28). Although the sample size and number of outcome events in this study were relatively small, the results suggest the usefulness of GNRI in predicting heart disease risk in middle-aged adults. The GNRI has mostly been studied in clinical settings; however, the results of this study can also be applied to middle-aged populations at the community level. Large-scale surveys are required to confirm the results of this study and elucidate the precise underlying mechanisms.

## 5. Conclusion

This study demonstrated that the GNRI score in middle-aged Korean adults, predicts adverse cardiovascular events later in life. Adding the GNRI score to the existing risk prediction model significantly increases its ability to predict cardiovascular events. The GNRI could be used as a practical tool to formulate routinely tested parameters for high-throughput screening of the long-term risk of CAD in the area of public health, which might support the prognostic stratification of high-risk community populations.

## Data availability statement

The data presented in this study are available on request from the corresponding author upon reasonable request.

## Ethics statement

The studies involving humans were approved by Institutional review board of Daegu University. The studies were conducted in accordance with the local legislation and institutional requirements. Written informed consent for participation was not required from the participants or the participants’ legal guardians/next of kin in accordance with the national legislation and institutional requirements.

## Author contributions

JP: Investigation, Writing – original draft, Writing – review & editing. SB: Conceptualization, Formal analysis, Funding acquisition, Investigation, Methodology, Project administration, Writing – original draft, Writing – review & editing.
